# Characterization of bovine and ovine basal-out and apical-out ileum organoids

**DOI:** 10.1098/rsos.250326

**Published:** 2025-07-30

**Authors:** Ambre F. Chapuis, Tanith Harte, Daniel R. G. Price, Marc N. Faber, William M. Anderson, Barbara Shih, Jayne C. Hope, Jo Moore, David Smith

**Affiliations:** ^1^ Moredun Research Institute, Penicuik, Scotland, UK; ^2^ The University of Edinburgh The Roslin Institute, Edinburgh, Scotland, UK; ^3^ Department of Biomedical and Life Sciences, Lancaster University, Lancaster, Lancashire, UK

**Keywords:** apical-out, basal-out, three-dimensional organoids, *in vitro* culture systems, LGR5+ stem cells, crypts, mucosal biology, mucosal surfaces, epithelial

## Abstract

Organoids are three-dimensional stem cell-derived structures that differentiate into multiple cell types. Their capacity to self-organize, coupled with the presence of diverse cell types, means that organoids resemble their organ of origin in architecture and function. Organoids from intestinal tissues have been extensively used as a three-dimensional model for *in vitro* studies of the gut. However, they typically self-organize with basal-out polarity when cultured in a three-dimensional extracellular matrix scaffold, presenting a hurdle for experiments that require access to the apical epithelial surface. Methods to invert the surface polarity of intestinal organoids have been reported, but little information exists on how this change of polarity impacts gene expression and cell populations present within the organoids. To address this knowledge gap, we modelled both polarity states in intestinal organoids from two different ruminant species. Apical-out organoids largely retained the same gene expression profile as basal-out organoids. Moreover, a combination of RNA-seq and immunohistochemistry analyses demonstrated the retention of specific markers of enterocytes, enteroendocrine, goblet and tuft cells present in organoids of both polarity states. This study presents a comprehensive validation of apical-out ileal organoids, providing supporting evidence for the utility of this model in experiments that require access to the apical surface.

## 1. Background

The field of organoid research has made significant progress in recent years, with the development of microscopic, self-organizing, multicellular cell culture models that closely resemble the *in vivo* tissues [1]. Moreover, the ability to cryopreserve and cultivate organoids in the laboratory over a relatively long period of time provides researchers with practical models that can be used to explore intricate biological questions with exceptional precision, as well as providing an ethical advantage towards reducing dependence on live animals for experimental biological research. Organoids can be developed from embryonic stem cells and induced pluripotent stem cells, or adult tissue-resident stem and progenitor cells [2–6]. The survival, proliferation and differentiation of these stem cells are driven through the use of culture media containing specific growth factors and inhibitors. For example, adult LGR5+ stem cell-containing crypts can be stimulated to form self-renewing intestinal organoids containing enterocytes, mucous-producing cells, Paneth cells, enteroendocrine cells, tuft cells and transit amplifying cells when cultivated in growth media containing combinations of the growth factors WNT3A, R-spondin, noggin, epidermal growth factor (EGF), fibroblast growth factor (FGF) and insulin-like growth factor (IGF) [7–10]. Initially, intestinal organoids were developed from human and rodent model stem cell types, with the development of organoids representing a diverse range of organs such as functional heart, mammary and tear duct organoids [11–15]. However, this species diversity has been extended in recent years, with organoids having now been developed for a range of mammalian, avian and reptilian species, including cattle, sheep, goat, pig, horse, cat, dog, bat, chicken and turtle [7,10,16–27].

Organoid models are not without their limitations. Under standard culture conditions, within an extracellular matrix (ECM) scaffold, organoid models of the gastrointestinal tract form with a luminal space. The epithelial surface facing this luminal core is typically the apical cell surface, with the basal cell surface presented on the outside of the organoid. This inherent polarity in organoids cultured within an ECM can make it difficult to study interactions and cellular biology that occur at the apical surface, as the basal surface can act as a barrier that needs to be overcome. For some studies, it is desirable to have access to the apical epithelial surface; examples include physiological studies of nutrient uptake, delivery of nanoparticles for regenerative medicine and studies of pathogen attachment and invasion at the apical surface [22,28–30].

Microinjection of material into the luminal core has been applied as a methodology to directly access the apical epithelial cell surface in a three-dimensional organoid model. However, the limitations of this method include the accumulation of cellular debris and mucus within the lumen, accessibility of specific equipment and training for performing microinjection and low experimental throughput [31,32]. An alternative methodology to directly access the apical surface involves inverting the polarity of organoids such that the apical surface is presented on the outside of the organoid [10,24,28,29]. Advantages of this approach include the ease with which polarity can be inverted, as this only requires thorough removal of the ECM, as well as higher throughput since the majority of organoids in a sample will become apical-out. However, while the inversion of polarity in three-dimensional gastrointestinal organoids has been previously reported, there has been no documented assessment on global molecular changes that might occur in an apical-out model. Therefore, the present study aims to provide a more complete characterization and validation of the apical-out organoid model by determining similarities and differences in the transcriptomic and cellular profiles of apical- versus basal-out intestinal organoids, using two ruminant species as models.

## 2. Methods

### 2.1. Animals

All bovine and ovine ileal tissues used in this study were derived from three−eight-month-old calves (Holstein-Friesian *Bos taurus*) and lambs (Texel cross *Ovis aries*). The presented research was performed following the 3Rs principles, particularly for the reduction of animals used in research. The animal tissues used for developing organoids in this study were opportunistically derived post-mortem from healthy control animals used in separate research trials performed at the Moredun Research Institute (MRI), UK.

### 2.2. Reagents and materials

A list of reagents and materials used in the present study, including suppliers and catalogue numbers, can be found in electronic supplementary material, table S1. This list includes culture media and supplements for the cultivation of organoids, fixation and antibody labelling reagents for immuno-histochemistry (IHC) analysis and kits for the isolation of RNA from organoids.

### 2.3. Isolation of intestinal crypts

Intestinal crypt isolation and subsequent organoid cultivation were carried out following the protocol described by Smith *et al.* [10]. Ten-centimetre segments of ileal tissue were harvested post-mortem and opened longitudinally with a sterile scalpel blade before gently scraping the epithelial cell surface with a sterile glass slide to remove the mucus layer. More pressure was then applied with the glass slide to lift the crypt-containing mucosal layer, which was transferred to a 50 ml Falcon tube containing 45−50 ml Hanks’ balanced salt solution (HBSS, containing 25 µg ml^−1^ gentamicin and 100 U ml^−1^ penicillin/streptomycin). Samples were centrifuged at 400*g* for 2 min, and the mucus layer was gently removed by aspiration. The pellet was then washed again in HBSS (containing 25 µg ml^−1^ gentamicin and 100 U ml^−1^ penicillin/streptomycin). This process was repeated until the HBSS media was found to be clear, and no apparent mucus layer was present. To release intestinal crypts from tissue, mucosal tissue pellets were resuspended in 25 ml of digestion medium (Dulbecco’s modified eagle medium (DMEM); high glucose (11574486; Gibco), 1% FBS, 20 µg ml^−1^ dispase (4942086001; Roche), 75 U ml^−1^ collagenase (C2674; Sigma-Aldrich), 25 µg ml^−1^ gentamicin and 100 U ml^−1^ penicillin/streptomycin) and incubated horizontally in a shaking incubator at 80 r.p.m. for 40 min at 37°C. The supernatant containing released crypts was then transferred to a sterile 50 ml Falcon tube and resuspended in 1−2 ml of organoid suspension medium (advanced DMEM/F12 medium containing 1X B27 supplement minus vitamin A, 25 µg ml^−1^ gentamicin and 100 U ml^−1^ penicillin/streptomycin). A 10 µl aliquot was observed under light microscopy to assess crypt integrity and to quantify crypts.

Approximately 200 intestinal crypts in 100 µl of organoid suspension medium were then added to 150 µl of BD Growth Factor Reduced Matrigel Matrix (356230; BD Biosciences), and 50 µl droplets were added to consecutive wells of a 24-well tissue culture plate (3524, Corning). Plates were incubated at 37°C, 5% CO_2_ for 15−20 min to allow the Matrigel to polymerize and then 550 µl of pre-warmed complete IntestiCult Growth Medium (mouse) (6005; STEMCELL Technologies) containing 500 nM Y-27632 (10005583; Cambridge Bioscience), 10 µM LY2157299 (15312; Cambridge Bioscience), 10 µM SB202190 (ALX-270-268-M001; Enzo Life Sciences) and gentamicin (50 µg ml^−1^) were added to each well. Plates were incubated at 37°C, 5% CO_2_ to allow organoids to develop, replacing complete IntestiCult medium every 2−3 days. Organoids were left to grow to maturity for 7−14 days before being passaged.

### 2.4. Organoid passage

IntestiCult media was removed from the cultured organoids and the Matrigel matrix was dissolved by replacement with 1 ml ice-cold advanced DMEM/F12. The resuspended organoids were transferred to a 15 ml Falcon tube, and the total volume of advanced DMEM/F12 was increased to 8 ml. Samples were centrifuged at 200*g* for 2 min. Organoid pellets were resuspended in 1 ml advanced DMEM/F12 medium (containing 1X B27 supplement minus vitamin A, 25 µg ml^−1^ gentamicin and 100 U ml^−1^ penicillin/streptomycin) and then mechanically disrupted by repeatedly pipetting (approximately hundred times) using a 200 µl pipette tip bent at a 90°. The organoid fragments were assessed by light microscopy, and 100 µl of fragments were then combined with Matrigel and plated into 24-well tissue culture plates as described in §2.1. Organoids were routinely monitored by phase microscopy.

### 2.5. Generation of apical-out organoids

**Figure 1.**
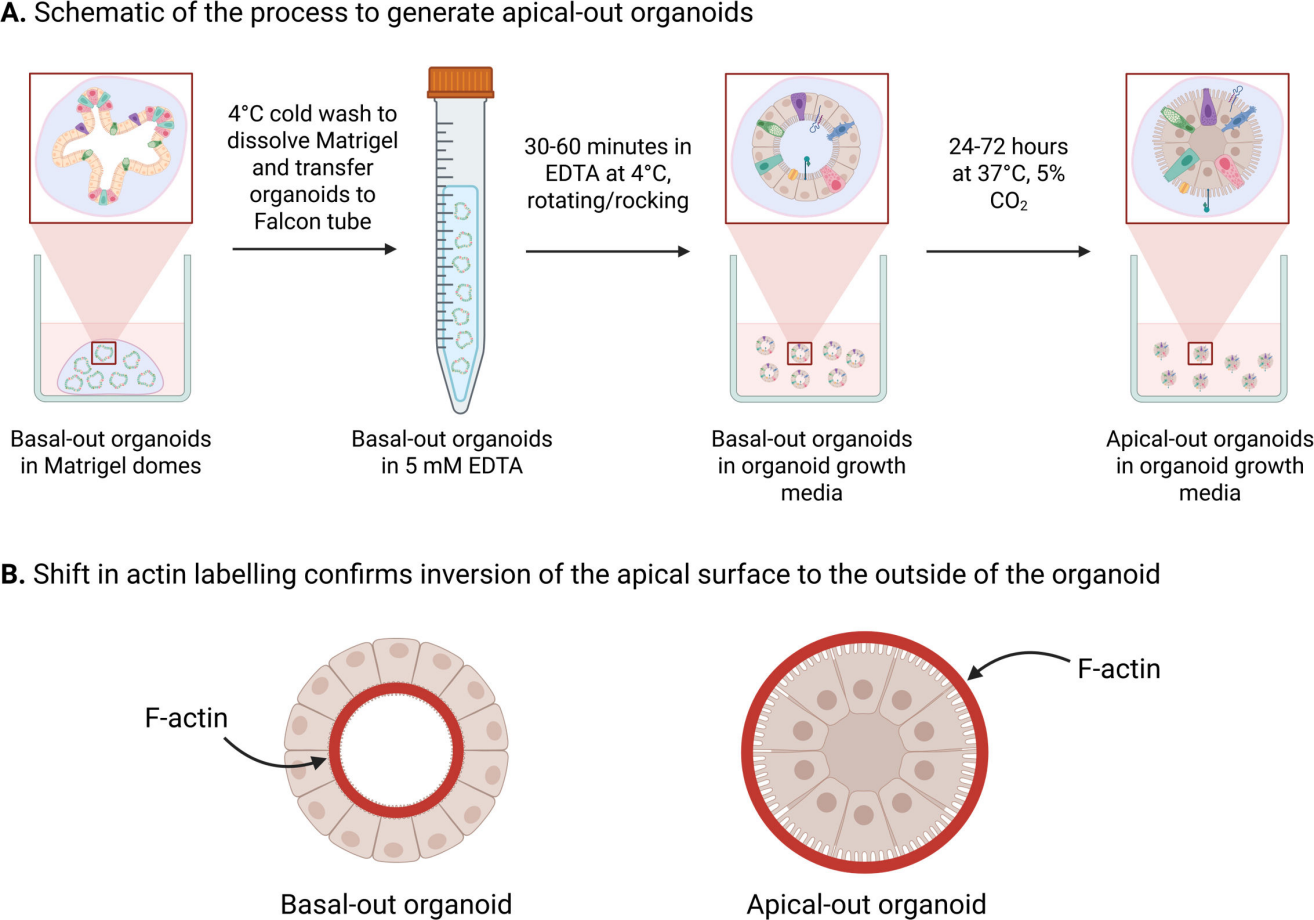
Making apical-out organoids in the laboratory. Schematic illustration summarizing the process for generating apical-out organoids from basal-out organoids (A). F-actin labelling is an effective method to demonstrate the polarity inversion, with the apical surface moving from inside the organoid (adjacent to the luminal space) to the outer organoid surface in apical-out organoids (B). Figure prepared using BioRender.

Epithelial polarity was inverted in ileal bovine and ovine organoids by following a previously published method for reverse polarity in human and sheep intestinal organoids [10,28,33]. Organoids were grown to maturity in Matrigel for 7 days. Organoid-containing Matrigel domes were then gently dissolved by removing the IntestiCult Organoid Growth Media and replacing it with 500 µl ice-cold 5 mM ethylenediaminetetraacetic acid (EDTA) in phosphate buffered saline (PBS), making sure not to rupture the organoids. The resulting suspension was transferred to a 15 ml Falcon tube that was subsequently filled with 14 ml of ice-cold 5 mM EDTA. Samples were placed on a rotator and mixed gently for 1 h at 4°C. Organoids were pelleted by centrifugation at 200*g* for 5 min at 4°C and the supernatant was removed. Pellets were resuspended in complete IntestiCult growth media (containing 500 nM Y-27632, 10 µM LY2157299, 10 µM SB202190 and gentamicin (50 µg ml^−1^)), with the addition of 10% advanced DMEM/F12 medium (containing 1X B27 supplement minus vitamin A, 25 µg ml^−1^ gentamicin and 100 U ml^−1^ penicillin/streptomycin). Resuspended organoids were transferred to the wells of eight-well glass chamber slides and incubated at 37°C, 5% CO_2_ for a period of 72 h. This process is summarized in figure 1. Apical-out and basal-out organoids are readily identifiable in culture by light microscopy. Therefore, in order to determine the consistency with which apical-out polarity reversal occurred within individual cultures, the number of apical- and basal-out organoids was counted after 48 h of suspension culture. Three individual experiments were performed for both bovine and ovine cultures.

### 2.6. Immunohistochemistry

*Whole-mount*. Organoids were fixed with cold 10% formalin for 20 min. Samples were then washed twice with immunofluorescence (IF) buffer (0.1% Tween 20 in PBS), media removed and then permeabilized using 0.1% TritonX-100 in PBS for 1 h at room temperature (RT), followed by three washes with IF buffer. Organoids were blocked using 1% bovine serum albumin (BSA) in IF buffer at RT for 30 min, followed by incubation with primary antibodies diluted in blocking solution overnight at 4°C. Primary antibodies used included polyclonal rabbit α-Ki67 (ab15580, Abcam, used at a 1 : 500 dilution) and α-POU2F3 (HPA019652, Sigma-Aldrich, used at 1 : 200 dilution). Rabbit IgG antibodies (Sigma-Aldrich) were used as isotype controls and were diluted at 1 : 5550. The following day, samples were washed three times in IF buffer, and the secondary antibodies were diluted in blocking buffer and incubated for 1 h at RT. The secondary antibodies used were goat anti-rabbit Alexa Fluor 488 (ab150081, Abcam, at 1 : 500 dilution). After washing the samples three times with IF buffer, nuclei and F-actin were stained with Hoechst 33258 (94403, Sigma-Aldrich, 1 : 200 dilution) and Phalloidin-iFluor (ab176756, Abcam, 1 : 1000), respectively, by incubation for 15 min at RT followed by three washes with IF buffer. Samples were mounted using ProLong Diamond antifade mountant (P36965, Thermo Fisher Scientific). Organoids treated only with their isotype controls (rabbit serum IgG) were negative for any labelling (data not shown).

*Paraffin embedded*. Organoids were paraffin embedded for IF and periodic acid-Schiff (PAS) labelling. Organoids were collected from three−four wells using ice-cold DMEM and pelleted by centrifugation at 400*g* for 5 min. The organoid pellet was resuspended in ice-cold 10% neutral-buffered formalin (NBF) and left to fix at RT for 30 min. After fixation, organoids were pelleted by centrifugation (using conditions described above) and washed with PBS to remove formalin. Organoids were then resuspended in 200 µl Epredia Histogel (12006697, Fisher Scientific) and placed into an embedding mould on ice for 20 min to set. The solidified histogel block was removed from the mould and placed into a specimen cassette for paraffin embedding.

Tissue for IHC/IF was collected at post-mortem and immediately placed into 10% NBF and then left to fix overnight at 4°C. Once fixed, NBF was replaced with 70% ethanol for storage until tissue was trimmed and placed into a cassette for paraffin embedding. After embedding, both tissue organoids were cut into 4 µm thick sections using a microtome and placed onto Epredia superfrost slides and then allowed to dry overnight at 25°C.

For IF staining, slides were deparaffinized and rehydrated using the following procedure: 3 min in xylene, 1 dip in xylene (fresh xylene for each change), 3 min in 100% ethanol, 3 min in 95% ethanol, 3 min in 70% ethanol and 3 min in tap water. Slides were then submerged in sodium citrate buffer (pH 6, 0.0874 M sodium citrate, 0.0126 M citric acid) and autoclaved at 90°C for 15 min. After cooling, slides were rinsed with PBS and then permeabilized for 10 min with 0.1% Triton X-100 in PBS. Blocking solution (10% BSA, 0.025% Tween 20 in PBS) was applied for 1 h at RT. Primary antibodies were then applied overnight at 4°C and diluted in blocking solution. Primary antibodies used were Rabbit-anti-chromogranin A (Novus Bio, NB120-15160, used at 1 : 500 dilution) and Rabbit-anti-POU2F3 (HPA019652, Sigma-Aldrich, used at 1 : 200 dilution). The following day, slides were rinsed three times with PBS, and secondary antibodies diluted in blocking buffer were applied and incubated for 1 h at RT. The secondary antibody used was goat-anti-rabbit IgG Alexa-Fluor 488 (A-11008, Thermo-Fisher, used at 1 : 1000 dilution). Slides were then rinsed three times with PBS and incubated with DAPI (D1306, Thermofisher, 1 mg ml^−1^ diluted 1 : 1000 in PBS) for 10 min. Finally, slides were rinsed three times with PBS, and coverslips were mounted with ProLong Gold antifade mounting media (P36930, Thermofisher) and allowed to set overnight at 4°C before imaging.

PAS staining was performed by deparaffinizing tissue as previously described, followed by oxidizing slides using 1% periodic acid for 5 min. Sections were rinsed in tap water for 5 min and then treated with Schiff’s reagent for 15 min. Next, slides were rinsed under running tap water for 20 min and then submerged in haematoxylin Z (RBA-4201-00A, Cellpath) counterstain for 3 min and rinsed in tap water until clear. Slides were then briefly dipped in 1% acid alcohol to remove excess haematoxylin and then nuclei blued using Scott’s tap water substitute for 30 s. Finally, slides were dehydrated using the reverse procedure for deparaffinization, then coverslips were mounted using DPX mount.

### 2.7. Microscopy

Fluorescent images of organoids were taken on a Zeiss Axiovert 200 M microscope using an AxioCam MR3, an Apotome 2 and a Colibri 7 LED light source, operating with Zen Blue (version 3.1). Z-stacks were taken using the Apotome grid for optical sectioning, generating five raw images per Z-plane and channel, which were subsequently processed into one image using phase correction and Fourier filtering to remove residual Apotome grid artefacts and deconvoluted to account for spherical aberration. Maximum intensity projections visualizing the *x*/*y*, *x*/*z* and *y*/*x* planes were generated for each organoid. Live imaging of organoids was performed on a Zeiss Axio Observer 7 inverted microscope using an Axiocam 705 and carrier transmitted-light illumination, operating on Zen Blue (v. 3.3). Plate overviews were generated using the Zeiss AI sample finder to generate tiled overviews of each chamber slide well. Environment controls were set to 37°C and 5% CO_2_ in line with normal culture conditions. Overview scans were captured every hour over 24 h, and individual tile areas were stitched using the Zen Blue stitching function, including edge detection and tile fusing.

### 2.8. Total RNA extraction from organoids

Total RNA was extracted from apical-out and basal-out organoids using a Qiagen RNeasy mini kit, which included an on-column DNAase I digest. Organoids were recovered from the Matrigel matrix with 1 ml ice-cold advanced DMEM/F12, and organoid pellets were resuspended in 350 µl RLT buffer (Qiagen) containing β-mercaptoethanol and stored at −70°C until extraction. Total RNA from each extraction was quantified using a NanoDrop™ One spectrophotometer, and RNA integrity was analysed on a Bioanalyzer 2100 instrument using an Agilent RNA 6000 Nano Kit (Agilent). All isolated RNA had a ribosome integrity number value greater than 7 and was stored at −70°C until RNA sequencing (RNA-seq) analysis.

### 2.9. RNA sequencing analysis

All library synthesis and sequencing was performed by BGI Genomics (Hong Kong, China). In brief, dual-indexed, strand-specific RNA-seq libraries were constructed from submitted total RNA samples. The barcoded individual libraries were pooled and sequenced on a DNBseq^TM^ G400 platform (paired-end, 2 × 150 bp sequencing), generating on average 30 million reads per sample (electronic supplementary material, table S2).

### 2.10. Bioinformatic and statistical analysis

Following sequencing, adaptors were trimmed from raw data and low-quality sequences removed using SOAPnuke (with the following parameters: -n 0.01 l 20 -q 0.4 --adaMR 0.25 --ada_trim --polyX 50 --minReadLen 150) [34]. Sequence reads were checked for quality using FastQC v. 0.11.9 [35]. Reads were pseudo-aligned to the *Bos taurus* transcriptome (ARS-UCD1.2, https://www.ensembl.org/Bos_taurus/Info/Index) and the *Ovis aries* transcriptome (Oar_v3.1; https://www.ensembl.org/Ovis_aries/Info/Index) using Kallisto v. 0.46.2 with default settings [36]. Due to the more complete annotation of bovine genes, mapping rates for both datasets were higher when mapped to the *Bos taurus* transcriptome; thus, these mappings were used for downstream analyses. A summary of read data and mapping rates is shown in electronic supplementary material, table S2. Estimated read counts generated by Kallisto were exported as a read count matrix, which was trimmed, removing genes with less than 10 read sum counts. Gene expression data were analysed by principal component analysis (PCA) using the pcaExplorer v. 2.23.0 R/Bioconductor package [37].

For RNA-seq analysis, Kallisto-generated estimated count data was imported into the DESeq2 package using the tximport package [38] and then normalized within the DESeq2 package using the median of ratios method ‘estimateSizeFactors()’ [39]. Differential expression analysis was made by performing two pairwise comparisons between the following datasets: bovine apical-out versus apical-out and ovine apical-out versus apical-out. Genes were considered differentially expressed with adjusted *p*-value < 0.01 and a log2 fold change < −2 or > 2. Volcano plots and heatmaps were generated using the R packages ggplot2 and pheatmap, respectively.

Gene set over-representation analysis was performed using the enrichGO function in R package clusterProfiler (version 3.1.8.1) [40] with the parameter set to OrgDb = org.Bt.eg.db, ont = BP or MF, pAdjustMethod = BH. Identified enriched GO (Gene Ontology) terms had a *p*-adjusted value < 0.05 after Benjamini–Hochberg correction for multiple testing.

To determine whether bovine and ovine ileal organoids (in both orientations) retained the expression of epithelial cell type markers associated with the ileum, we identified and analysed the expression of a set of ileum-specific gene markers. The set of ileal gene markers was derived from a single-cell sequencing study of the mouse ileum [41]. This dataset contains markers for the following cell types: enterocyte cells (71 genes), enteroendocrine cells (EEC; 37 genes), goblet cells (72 genes), Paneth cells (2 genes) and tuft cells (83 genes) [41]. Orthologues of these marker genes were extracted from the *Bos taurus* transcriptome using gene symbols through BioMart. Expression of each of the retrieved marker genes was plotted as a heatmap using the R package pheatmap.

## 3. Results

### 3.1. Inversion of ileum organoid polarity from basal-out to apical-out orientation

**Figure 2.**
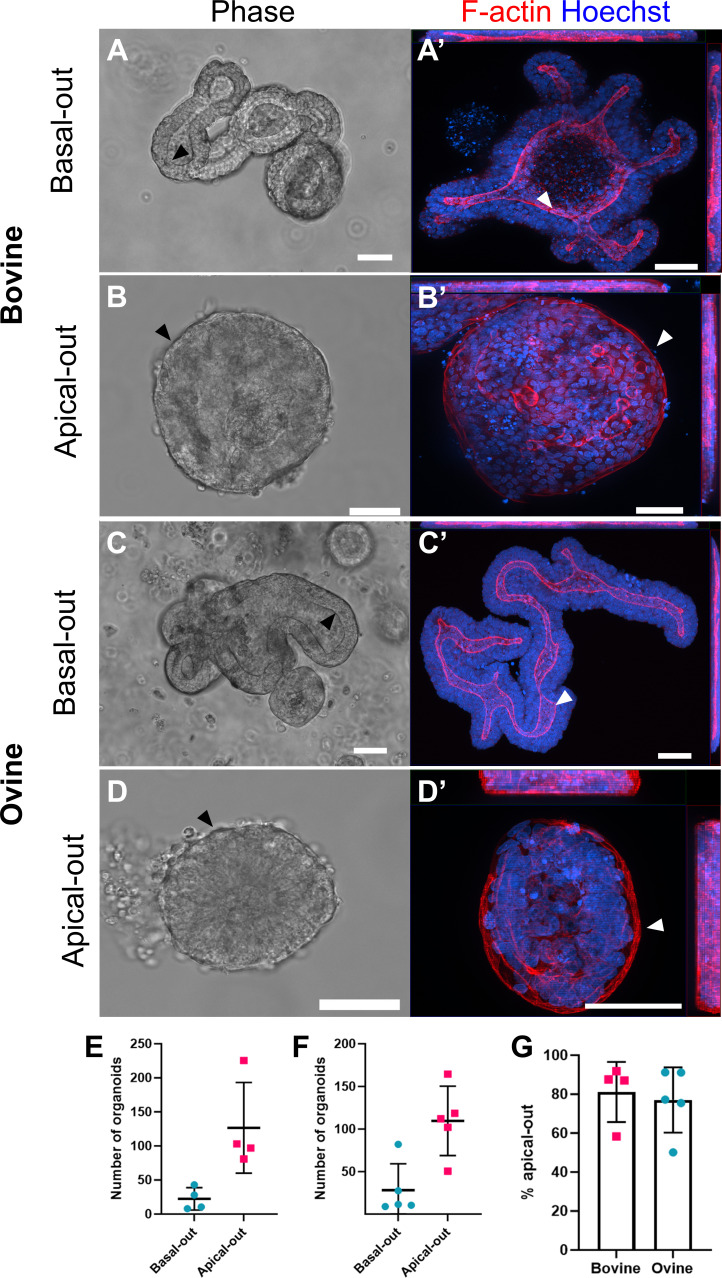
Representative images of bovine and ovine ileal organoids in basal-out and apical-out orientations. Bovine and ovine intestinal organoids in basal-out orientation (A, A′, bovine; C, C′, ovine) and in apical-out orientation (B, B′, bovine; D, D′, ovine). Organoid phase contrast images (panels A–D) and orthogonal maximum intensity projection images of different planes (*x*/*y*, *x*/*z* and *y*/*z*) labelled with F-actin (red) and nuclear DNA (blue) (panels A′–D′). White and black arrows indicate the F-actin border associated with the apical surface. Scale bars = 50 µm. Comparison of the number of basal-out organoids and apical-out organoids in bovine (panel E) and ovine (panel F) species. Each symbol represents the mean number of organoids from an individual experimental replicate. Percentage of apical-out ileal organoids (bovine and ovine) after 48 h of media suspension culture (G).

The epithelial cell polarity of bovine and ovine ileal organoids was flipped from a basal-out orientation to apical-out by dissolving Matrigel domes containing 7-day cultured organoids with ice-cold 5 mM EDTA. Released organoids were incubated on a platform rotator at 4°C for 1 h to thoroughly remove Matrigel, followed by a gentle centrifugation and resuspension in organoid growth media. Note that organoids incubated in EDTA at RT were not viable, with organoids collapsing within 24 h (data not shown). The location of the apical surface in basal-out and flipped apical-out organoids was confirmed by both phase contrast microscopy and immunofluorescent labelling of F-actin filaments (figure 2). In basal-out ileal organoids grown in Matrigel, the apical epithelial surface is located on the luminal surface of bovine (figure 2A,A’) and ovine (figure 2C,C’) organoids. Conversely, following EDTA treatment and 24 h of incubation in growth media in suspension, the apical epithelial surface was flipped to the outer organoid surface, visible as a solid F-actin positive ring on the external surface in both bovine (figure 2B,B’) and ovine (figure 2D,D’) ileal organoids. Moreover, apical-out organoids were immediately apparent in normal culture through their loss of a luminal compartment at their centre and a fan-like appearance in the outer epithelial cell layer, whereby the boundary between cells is readily visible by light microscopy (figure 2B,D). Since the inversion of epithelial polarity was demonstrated after 24 h of suspension culture, organoids were live imaged over a 24 h period following their removal from Matrigel domes and washing with EDTA. Time-lapse imaging revealed that organoid inversion occurred after 12 h for bovine and 10 h for ovine organoids in cultures performed in tandem (electronic supplementary material, S1). We also observed that cell debris was discharged from organoids during this period of inversion and that organoids became smaller in size following inversion (electronic supplementary material, S1).

Next, we sought to determine the efficiency of organoid inversion, i.e. the proportion of basal-out organoids that inverted to an apical-out polarity when treated with EDTA and cultured in suspension. After 48 h of organoid culture in suspension media, we found that 81.2% (±15.4%) of bovine organoids became apical-out and 77% (±16.8%) of ovine organoids became apical-out (figure 2E–G).

### 3.2. Retention of proliferating and differentiated cell types in apical-out organoids

**Figure 3.**
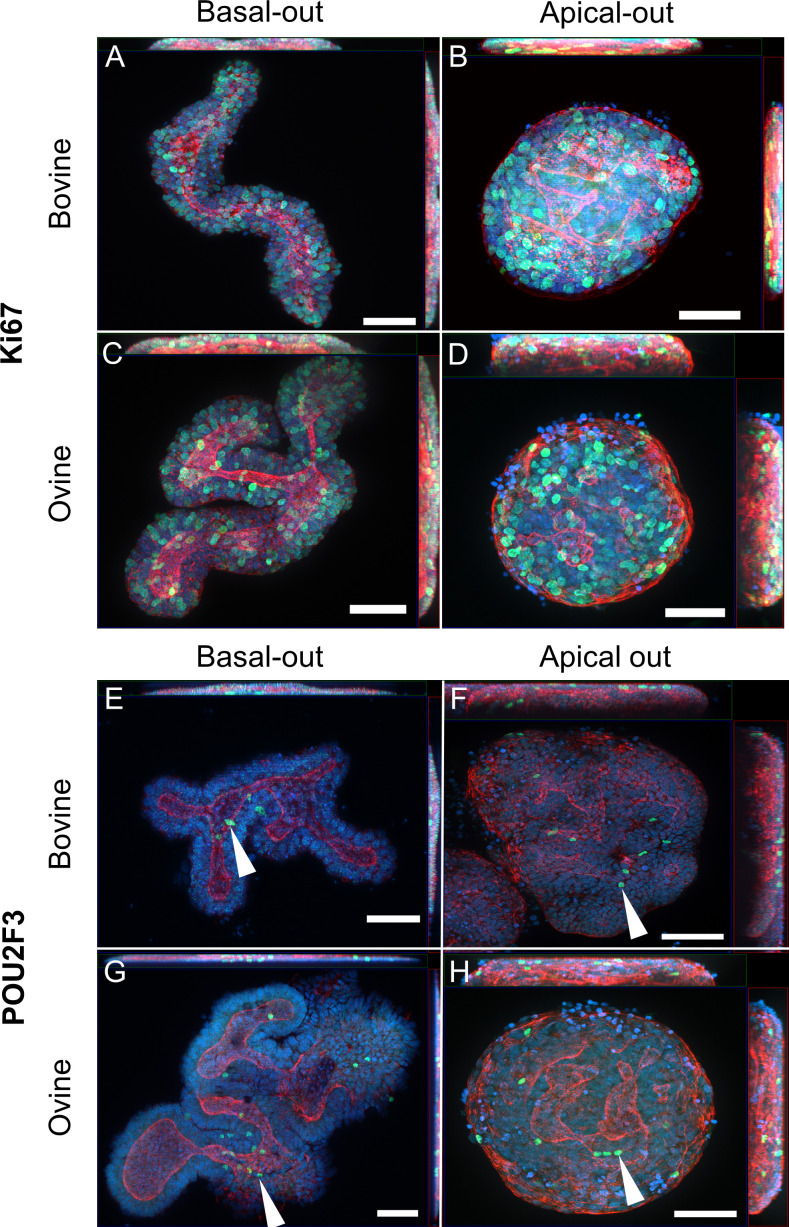
Cell marker analysis of basal-out and apical-out bovine and ovine ileal organoids. Representative bovine and ovine intestinal organoids in basal-out orientation (A and E, bovine; C and G, ovine) and apical-out orientation (B and F, bovine; D and H, ovine). Organoids from both species were fixed at 72 h post-flipping (for apical-out organoids) and a matched time point for basal-out organoids. Orthogonal maximum intensity projection images of different planes (*x*/*y*, *x*/*z* and *y*/*z*) in organoids stained for F-actin (red), nuclear DNA (blue), Ki67 (green, panels A–D) and POU2F3 (green, panels E–H). Examples of POU2F3+ cells are indicated with white arrowheads. Scale bars = 50 µm.

Prior to undertaking a broad transcriptomic analysis of the basal-out versus apical-out organoids, whole mount organoids were probed for the marker proteins Ki67 (proliferation marker) and POU2F3 (tuft cell marker, used as a proxy for a differentiated cell type) to generally confirm both proliferating and differentiated cell types were present in the organoids in both orientations. Basal-out ileal organoids (both bovine and ovine) contained a large number of proliferating Ki67+ cells (figure 3A,C), and this marker was retained when bovine and ovine ileal organoids were flipped to an apical-out orientation (figure 3B,D). Several POU2F3+ cells were detected in bovine and ovine organoids, both their basal-out and apical-out orientations, indicating that differentiated tuft cells were present in these organoids (bovine: figure 3E,F; ovine: figure 3G,H).

### 3.3. Bovine and ovine apical-out organoids retain a similar gene expression profile to basal-out organoids

**Figure 4.**
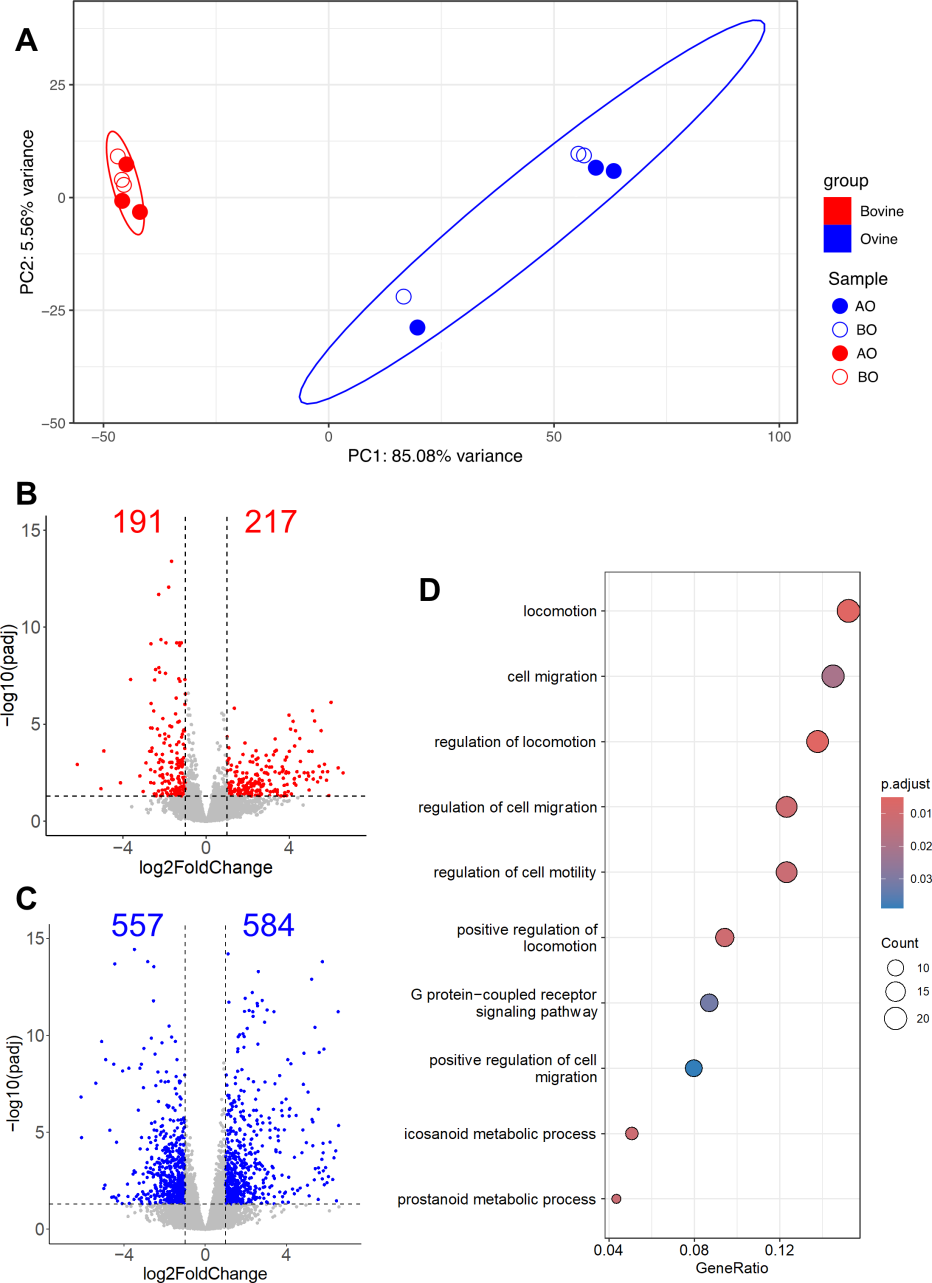
(A) PCA of the top 500 most variant genes (of genes ranked by inter-sample variance) in bovine and ovine ileal organoids. Sample type is indicated in the key and includes bovine (red) and ovine (blue) ileal organoids in basal-out and apical-out orientations. For each sample type, *n* = 3, and ellipses indicate 95% confidence intervals for each cluster. (B and C) Volcano plots showing differentially expressed genes (DEGs) in (B) bovine ileal and (C) ovine ileal organoids in basal-out versus apical-out orientations. The number of differentially regulated genes (log2 fold-change > 1; adjusted *p*‐value < 0.05) is shown in each plot. (D) Dot plot visualization of the top 10 enriched GO terms of up-regulated DEGs in ovine ileal apical-out organoids. The colour of the dots represents the *p*-value adjusted by Benjamini–Hochberg correction for each enriched GO term identified by Fisher’s exact test using the enrich GO function in the R package clusterProfiler, and the size of the dot represents the number of genes enriched in the total gene set.

To investigate whether the apical-out organoids displayed the array of cell types characteristically present in the gut and retained an overall gene expression profile similar to basal-out organoids, RNA-seq analysis was performed on both bovine and ovine basal-out and apical-out organoids. Expression profiles of the 12 organoid samples were initially analysed by PCA. Analysis of the top 500 most variable genes from bovine and ovine apical- and basal-out organoids indicated clustering of samples based on the species the organoids were derived from, with bovine and ovine organoids forming two distinct statistically different clusters (figure 4A). In bovine samples, apical-out and basal-out organoids clustered closely together, indicating an overall preservation in the global gene expression of the organoids (figure 4A). For the ovine ileal organoids, greater levels of heterogeneity were observed, but there was no separation (in the first two principal components) of ovine ileal organoids in basal-out and apical-out orientations (figure 4A).

Next, to identify differentially expressed genes (DEGs) in bovine and ovine organoids in basal-out and apical-out orientations, we performed two pairwise comparisons between basal-out and apical-out organoids from both species. In bovine organoids, we identified 408 DEGs (217 genes up-regulated and 191 genes down-regulated in apical-out versus basal-out organoids) (figure 4B). In ovine organoids, we identified 1141 DEGs (584 genes up-regulated and 557 genes down-regulated in apical-out versus basal-out organoids) (figure 4C). A full list of DEGs for bovine and ovine ileal organoid comparisons is presented in electronic supplementary material, tables S3 and S4, respectively. To get further biological insight into these DEGs, we performed over-representation analysis of GO terms associated with up- and down-regulated DEGs. Given the low number of DEGs identified in the bovine comparison, no significantly enriched GO terms were identified. However, for the ovine organoids, following flipping of the organoids to an apical-out orientation, terms associated with cell migration and motility were identified, along with a small number of genes associated with changes in metabolic processes (figure 4D).

**Figure 5.**
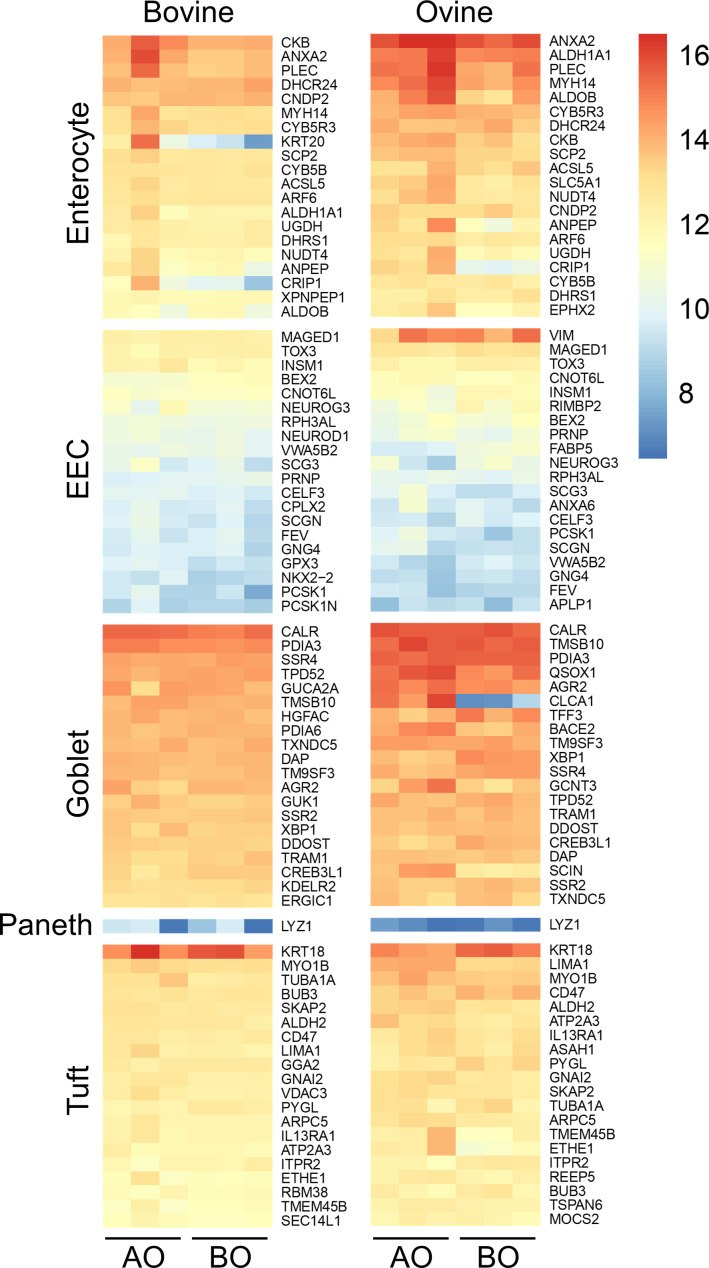
Transcription profiles of cell-type markers in bovine and ovine ileal organoids in basal-out and apical-out orientations. The heatmaps for bovine and ovine ileal organoids show the expression level of gene markers from specialized epithelial cell types, including enterocytes, enteroendocrine cells (EECs), goblet cells, Paneth cells and tuft cells. Sample types include bovine and ovine ileum organoids in basal-out (BO) and apical-out (AO) orientations. For each organoid type, *n* = 3 biological replicates. Colours in the heatmap indicate the levels of expression: red (high) and blue (low).

To determine whether bovine and ovine ileal organoids (in both orientations) retained the expression of epithelial cell type markers associated with the ileum, we identified and analysed the expression of a set of ileum-specific gene markers. Ileal gene marker sets were derived from a previous single-cell sequencing study of the mouse ileum [41]. This dataset contains markers for the following cell types: enterocyte cells (71 genes), enteroendocrine cells (EEC; 37 genes), goblet cells (72 genes), Paneth cells (2 genes) and tuft cells (83 genes) [41]. From this gene set, the majority of genes were identified in the bovine transcriptome, and the expression of these markers was conserved in bovine and ovine organoids in both orientations. First, this reaffirms that ileal organoids express markers of diverse epithelial cell types found in the ileum, and second, this confirms that the expression of these markers is retained in ileal organoids in both basal-out and apical-out orientations. The top 20 most highly expressed marker genes for each cell type for bovine and ovine organoids are shown in figure 5, and a full list of expressed genes is available in electronic supplementary material, tables S5 (bovine) and S6 (ovine). The majority of identified ileal cell-type markers were expressed at similar levels in ovine and bovine ileal organoids in both orientations. However, some gene markers were differentially expressed in apical-out versus basal-out organoids. Examples include up-regulation of enterocyte marker genes in ovine and bovine organoids in apical-out versus basal-out orientations, e.g. *KRT20* and *CRIP1* in bovine and *CRIP1* and *CLCA1* in ovine.

In summary, ileal organoids derived from bovine and ovine tissue express markers of ileal epithelial cell types. In addition, flipping organoids from basal-out to apical-out orientation results in relatively minor changes to the global expression profiles and flipped (apical-out) organoids retain the expression of ileal epithelial cell-type markers.

### 3.4. Bovine and ovine apical-out intestinal organoids retain diverse epithelial cell types

**Figure 6.**
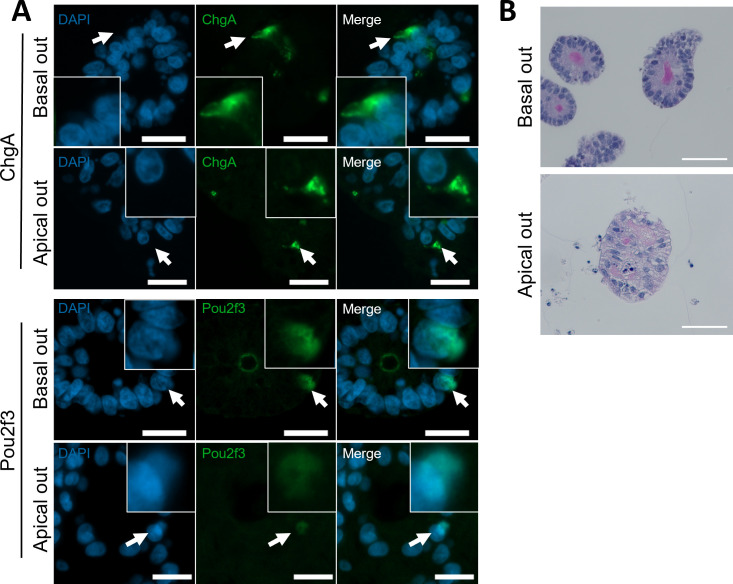
Expression of specialized cell types in bovine ileal organoids in basal-out and apical-out orientations. (A) Representative immunofluorescent antibody labelling against cell-type specific markers for EECs (CgA, A-top) and tuft cells (POU2F3, A-bottom) of basal-out and apical-out bovine organoids. Cell-specific labelling shown in green, and nuclear (DAPI) labelling shown in blue. Arrows point to individual cells showing positive staining, emphasized in the inset images. (B) Representative PAS staining for mucus produced by goblet cells in basal-out (top) and apical-out (bottom) bovine organoids. Scale bars = 20 µm.

**Figure 7.**
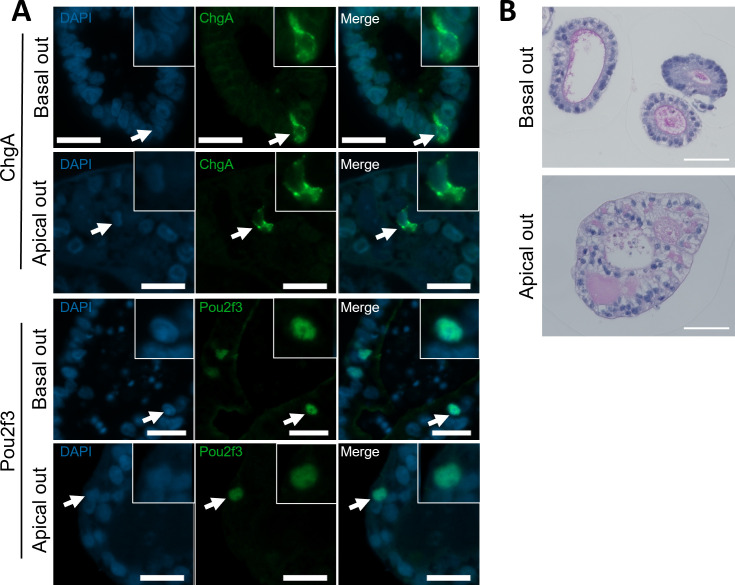
Expression of specialized cell types in ovine ileal organoids in basal-out and apical-out orientations. (A) Representative immunofluorescent antibody labelling against cell-type specific markers for EECs (CgA, A-top) and tuft cells (POU2F3, A-bottom) of basal-out and apical-out ovine organoids. Cell-specific labelling shown in green, and nuclear (DAPI) staining shown in blue. Arrows point to individual cells showing positive staining, emphasized in the inset images. (B) Representative PAS staining for mucus produced by goblet cells in basal-out (top) and apical-out (bottom) orientation. Scale bars = 20 µm.

RNA-seq analysis confirmed that apical-out organoids retained the transcription profiles of genes associated with specialized epithelial cells (figure 5). We therefore sought to confirm the presence of mucus-producing, enteroendocrine (EEC) and tuft cells in basal-out and apical-out organoids by IF microscopy. Using antibodies against cell-specific markers, we showed that basal-out and apical-out organoids for both species contained POU2F3-positive tuft cells and chromogranin A (CgA)-positive enteroendocrine cells (figure 6A, bovine, and figure 7A, ovine). As expected, the POU2F3 (a tuft cell-specific transcription factor) localized to the nucleus of stained organoid cells, while the CgA (found in secretory vesicles of EEC cells) localized to regions of the cell cytoplasm (figures 6 and 7). The staining patterns observed in organoids reflect localizations that were performed with ileum tissue sections from cattle and sheep (electronic supplementary material, figure S2). Next, to confirm the presence of mucus-producing goblet cells, we performed PAS staining for mucus (figures 6B and 7B). These cells were expressed at a similar rate in both apical-out and basal-out organoids from both species and remain representative of the numbers of these cells expressed in individual crypts from tissue (electronic supplementary material, figure S2).

## 4. Discussion

Organoids have emerged as invaluable tissue culture models that facilitate precision-based research of discrete tissue sites. However, the majority of three-dimensional organoid models form with a basal-out cellular polarity. This basal-out organoid structure poses a practical challenge regarding direct access to the apical surface in experiments. Methods applied to directly access the apical surface (e.g. for organoid challenge with a pathogen) include fragmentation of organoids, single cell digest (followed by co-incubation), microinjection and two-dimensional culture. However, these methods have limitations, including low throughput, requirement for specialist equipment and training, damage or dissociation of the organoid culture and/or loss of three-dimensional organoid architecture [42–46]. These factors led to the development of apical-out intestinal organoids, first established by Co *et al.*, whereby it was demonstrated that human ileal organoid polarity could be inverted with the apical epithelial surface facing outwards [28,33]. We subsequently showed this was possible with ovine gastric and intestinal organoids [10], and both studies demonstrated that an apical-out conformation permits direct access to the epithelial cell surface, e.g. for pathogen challenge. Other studies that have involved the use of apical-out organoids have also demonstrated their utility as models for pathogen challenge, host–microbe interactions, drug absorption and nutrient uptake [22,47–50]. The localization of the apical surface on the outside of organoids makes such types of experiments more practical, as researchers can simply introduce pathogens or treatments into the culture medium of the organoids without the need for any specialist equipment. The importance of the apical surface orientation in pathogen challenge, drug and vaccine treatment or nutrient uptake experimental models is an important factor to consider, since the precise localization of certain host proteins is limited to specific cell surfaces. For example, toll-like receptors, G protein-coupled receptors, succinate transporters and other molecular transporters (e.g. aquaporins, CFTR and CLCA1) have specific cell surface localizations and directionality throughout different regions of the gastrointestinal tract [51–55]. Therefore, it is important that the organoid model mimics the natural orientation found *in vivo* to properly assess the function of specific host surface-exposed antigens and their interactions with other molecules, ligands, proteins and pathogens.

Established methods used to demonstrate a shift in apical surface polarity include brightfield microscope, F-actin labelling, ZO-1 tight junction labelling and fluorescein isothiocyanate (FITC)-dextran permeabilization/exclusion [28,33]. Here, we used a combination of brightfield microscopy and F-actin labelling as an effective method to successfully demonstrate the shift in the polarization of the apical surface in ruminant intestinal primary organoids. However, while the utility of apical-out organoids in experimental models has been clearly established, the extent to which host cell diversity and gene expression profiles are retained remains unclear. Here, we present an in-depth validation of apical-out organoids. We demonstrate that in both bovine and ovine ileal organoids, cell diversity is retained following a shift from a basal-out to an apical-out conformation. This was demonstrated by the retention of cell type-specific markers for enterocytes, enteroendocrine, Paneth, goblet and tuft cells in transcriptomic experiments and by antibody labelling of the tuft cell transcription factor POU2F3, the enteroendocrine cell marker ChgA and PAS stain for mucus produced by goblet cells. Moreover, while DEGs were detected in RNA-seq datasets for bovine and ovine apical- versus basal-out organoids, the number of genes was relatively low, particularly for bovine ileal organoids, whereby no GO terms were found to be enriched or diminished in apical-out organoids. A greater number of genes were differentially expressed in ovine ileal organoids; however, GO term analysis of DEGs in ovine samples showed no processes to be down-regulated. Moreover, predominantly up-regulated terms indicated that cell locomotion, migration and motility-related genes were enriched in ovine apical-out organoids, which could be reflective of cells actively flipping to an apical-out conformation.

We do note the specific up-regulation of *CLCA1* and *CRIP1* in apical-out organoids, which could be indicative of cell stress [56–58]. In our experience, gastrointestinal apical-out organoids can typically persist for no more than 5−7 days in suspension culture, and therefore, the enrichment of these cell markers could be indicative of stress starting to occur 48−72 h into a suspension culture. Therefore, apical-out organoids should be used for endpoint experiments with a limited period of suspension culture so as not to confound stress responses, particularly in a challenge experiment whereby a response to challenge could be confounded with an inherent stress response in apical-out organoids following prolonged suspension culture. This is in line with findings elsewhere for canine apical-out intestinal organoids [59]. However, we have also observed that apical-out organoids can be re-embedded into a three-dimensional scaffold (e.g. Matrigel) and will revert to a basal-out polarity, from which longer-term culturing and passage can be sustained (data not shown).

## 5. Conclusions

In summary, this report represents a comprehensive characterization and validation of apical-out intestinal organoids. We found that cell type diversity is retained in apical-out intestinal organoids derived from sheep and cattle ileal crypts and that gene expression profiles are relatively similar between apical- and basal-out organoids. This validation supports the utility of apical-out organoids for experiments in which direct access to the apical surface is key to resemble an experimental system mimicking the lumen of the mammalian gut, such as in host–pathogen interaction studies, drug uptake and nutrient treatment-based experiments.

## Data Availability

The RNA-seq datasets generated in this study are available at the NCBI Sequence Read Archive (SRA) Database under the project accession number: PRJNA935267. Supplementary material is available online [60].
